# A murine oral model for *Mycobacterium avium* subsp. *paratuberculosis* infection and immunomodulation with *Lactobacillus casei* ATCC 334

**DOI:** 10.3389/fcimb.2014.00011

**Published:** 2014-02-05

**Authors:** Meagan A. Cooney, James L. Steele, Howard Steinberg, Adel M. Talaat

**Affiliations:** ^1^Laboratory of Bacterial Genomics, Department of Pathobiological Sciences, University of Wisconsin-MadisonMadison, WI, USA; ^2^Food Science, University of Wisconsin-MadisonMadison, WI, USA; ^3^Department of Food Hygiene, Cairo UniversityCairo, Egypt

**Keywords:** pratuberculosis, oral model, probiotics, animal model, vaccine development

## Abstract

*Mycobacterium avium* subsp. *paratuberculosis* (*M. paratuberculosis*) the causative agent of Johne's disease, is one of the most serious infectious diseases in dairy cattle worldwide. Due to the chronic nature of this disease and no feasible control strategy, it is essential to have an efficient animal model which is representative of the natural route of infection as well as a viable treatment option. In this report, we evaluated the effect of different doses of *M. paratuberculosis* in their ability to colonize murine tissues following oral delivery and the ability of *Lactobacillus casei* ATCC 334, a nascent probiotic, to combat paratuberculosis. Oral inoculation of mice was able to establish paratuberculosis in a dose-dependent manner. Two consecutive doses of approximately 10^9^ CFU per mouse resulted in a disseminated infection, whereas lower doses were not efficient to establish infection. All inoculated mice were colonized with *M. paratuberculosis*, maintained infection for up to 24 weeks post infection and generated immune responses that reflect *M. paratuberculosis* infection in cattle. Notably, oral administration of *L. casei* ATCC 334 did not reduce the level of *M. paratuberculosis* colonization in treated animals. Interestingly, cytokine responses and histology indicated a trend for the immunomodulation and reduction of pathology in animals receiving *L. casei* ATCC 334 treatment. Overall, a reproducible oral model of paratuberculosis in mice was established that could be used for future vaccine experiments. Although the *L. casei* ATCC 334 was not a promising candidate for controlling paratuberculosis, we established a protocol to screen other probiotic candidates.

## Introduction

*Mycobacterium avium* subspecies *paratuberculosis* (*M. paratuberculosis*) is the causative agent of Johne's disease characterized by chronic granulomatous enteritis in ruminants (Collins, 2011). Dairy cattle are the primary species affected by this disease with a herd-level prevalence of 91% in the USA (Lombard et al., 2013). Because of the chronic nature of Johne's disease and difficulty to detect *M. paratuberculosis*, available control measures are limited. The currently available vaccines are only able to reduce fecal shedding of the bacterium, but do not prevent the development of infection (Kalis et al., 2001; Sweeney et al., 2009). Because *M. paratuberculosis* is a worldwide problem in cattle and a suspected agent for human Crohn's disease (Hermon-Taylor and Bull, 2002; Divangahi et al., 2008), alternative treatment options are needed to control Johne's disease in dairy herds. Recently, various strains of lactic acid bacteria (LAB), have been shown to inhibit the growth of *M. paratuberculosis* in fermented food (Van Brandt et al., 2011), providing the potential for its use to control infection in a suitable host. Current animal models for Johne's disease pathogenesis and vaccine studies are either inefficient (e.g., the intraperitoneal infection of mice) or expensive (e.g., the cow or goat models) (Hines et al., 2007). Developing a reproducible, inexpensive animal model that mimics the natural route of infection and employing such model to identify a potential control strategy is the main objective of this report.

Many strains of LAB have been shown to have probiotic properties (Hart et al., 2003; Ghadimi et al., 2010). The World Health organization defines probiotics as “live microorganisms which when administered in adequate amounts confer a health benefit to the host” (FAO/WHO report, 2002). Generally, probiotics are regarded as generally recognized as safe (GRAS) organisms (Van Brandt et al., 2011) which would facilitate their use to control Johne's disease in farm animals. Earlier studies on LAB suggested their ability to exclude pathogens (Neu, 2007), modulate the systemic and mucosal immunity (Matsuzaki and Chin, 2000; Ghadimi et al., 2010), improve the epithelial barrier function (Corr et al., 2009), reduce inflammatory responses (Yan et al., 2011), and finally, act an antimicrobial agent (Dobson et al., 2011). In most of these studies, mice were used to evaluate the performance of LAB. For Johne's disease, several animal models have been employed, but the most extensively studied model has been the murine model. Although this model has its limitations of lacking clinical signs of Johne's disease, such as severe intestinal lesions and diarrhea (Hines et al., 2007), we were able to produce histological and immunological patterns similar to those displayed in ruminants (Shin et al., 2006). Comparing different routs of murine infection with *M. paratuberculosis*, the intraperitoneal (IP) injection was able to induce reproducible infection in all inoculated animals. Alternatively, when the oral route was used, mouse infection was only established in approximately 58% of animals (Mutwiri et al., 1992; Veazey et al., 1995). Because the oral route is the natural route of infection in the target host (cattle) (Chacon et al., 2004), we decided to optimize the murine oral model and examine the potential of use of LAB to control paratuberculosis.

To evaluate the efficacy of LAB ATCC 334 against *M. paratuberculosis* infection, we first established a reproducible murine model that closely mimics the natural pathogenesis of *M. paratuberculosis* following oral inoculation (10^9^ CFU/animal). Infections were detected in at least one organ of all inoculated mice in addition to the development of *M. paratuberculosis*-specific cell-mediated immunity. Interestingly, LAB ATCC 334 treatments were able to modulate host immunity and reduce the pathology of murine paratuberculosis. Whether such findings could be reproduced in a ruminant model (e.g., goats) of Johne's disease remains to be examined and could provide a novel approach to control this disease in dairy herds.

## Materials and methods

### Bacterial strains and culturing conditions

*M. paratuberculosis strain* K10, first passaged through a BALB/c mouse and isolated from the mesenteric lymph node was grown at 37°C in Middlebrook 7H9 broth (Difco, Sparks, MD) supplemented with 10% ADC (2% glucose, 5% BSA fraction V, and 0.85% NaCl, 0.5% glycerol, 0.05% Tween 80, and 2 μg/ml mycobactin J (Allied Monitor, Fayette, MO) (Hsu et al., 2011). For animal inoculations, bacterial cultures were harvested at an optical density at 600 nm of 1.0, washed in phosphate-buffered saline (PBS), and resuspended in 1/10 volume of PBS. To enumerate bacteria, serial dilutions of *M. paratuberculosis* cultures were plated on supplemented Middlebrook 7H10 agar (Difco, Sparks, MD). Agar plates for enumerating colonization in animal tissues were also supplemented with 5 mg/ml vancomycin, 30 mg/ml amphotericin B, and 10 mg/ml nalidixic acid to reduce bacterial and fungal contamination (Wu et al., 2007).

The LAB ATCC 334 was fed daily to mice undergoing therapeutic treatment. Cultures were grown in 5 ml MRS broth (Difco, Sparks, MD) for 24 h at 37°C in stagnant conditions after which 1/100 of the culture was then transferred into fresh media and grown for 18 h (Jim Steele-personal communication). Cultures were then pelleted through centrifugation, washed 1× with PBS and resuspended in 1/10 volume in skim milk for storage at −20°C until use. The strain used for this study was transformed with plasmid pTRKH2 (Stephenson et al., 2011), which encodes an erythromycin resistance marker that could be used to quantify LAB ATCC 334 doses on MRS agar (Difco, Sparks, MD) (2.5 μg/ml erythromycin). For feeding trials, the strain survival at room temperature was evaluated by suspending a culture in water and incubation at room temperature for 1 week, during which time daily serial dilutions were taken to evaluate its viability.

### Animal infections with *M. paratuberculosis*

Female BALB/c mice were purchased from Harlan Laboratories (Indianapolis, IN) at 3 weeks of age, housed in filtered screened shoe box plastic cages (cleaned every other day) in a pathogen-free environment, and provided with food and water *ad libitum* according to the protocol approved by the Institutional Animal Care and Use Committee, University of Wisconsin-Madison. In the first trial, mice (*n* = 10) were inoculated with a low infection dose of *M. paratuberculosis* via oral gavage on two consecutive days. Bacterial inocula were given to one group at 10^4^ CFU/mouse and the second group at 10^5^ CFU/mouse per dose each day. Mice were sacrificed at 6 weeks post infection. At time of sacrifice, samples from the liver, spleen, mesenteric lymph node and intestines were collected for bacterial, histopathological, and immunological examination (Shin et al., 2006). Since samples were divided to test the aforementioned parameters, the possibility of heterogeneous distribution of *M. paratuberculosis* needs to be taken into account during interpretation of results. In the second trial, groups of mice (*n* = 20) were inoculated with a high infection dose (10^9^ CFU/mouse/day) of *M. paratuberculosis* via oral gavage on two consecutive days (Veazey et al., 1995). Mouse groups (*n* = 3–5) were sacrificed at 1 day, 3, 6, 12, and 24 weeks post infection. At time of sacrifice, samples from the liver, spleen, mesenteric lymph node and intestines were collected for bacterial, histopathological, and immunological examination. Fecal samples were also collected throughout the duration of the study to monitor bacterial shedding. Fecal samples were decontaminated by resuspension in 0.75% Hexadecylcetylpyridinium chloride (HPC) (Sigma-Aldrich), at a 1:10 ratio and standing for 24 h at room temperature (Stabel, 1997). Supernatant was then removed and centrifuged (3000 × g) for 10 min. Pellets were washed with PBS and resuspended in fresh PBS to be used for quantification by serial dilution plating on 7H10 agar. Samples were conducted in duplicate from a pooled sample of feces.

Tissue sections collected for histopathology were preserved in 10% neutral buffered formalin before being embedded in paraffin, cut into 4- to 5-μ m sections, and stained with hematoxylin and eosin or the Ziehl-Neelsen method for acid-fast staining. Tissue sections were scored by a trained pathologist blindly, based on severity and extent of inflammatory lesions (normal, lymphocytic inflammation, granulomatous inflammation, or granuloma) (Hsu et al., 2009).

## Feeding trials of *L. casei* ATCC 334

The *L. casei* ATCC 334 feeding trials included a preventative trial, in which ATCC 334 was fed before *M. paratuberculosis* challenge, a therapeutic trial, in which ATCC 334 was fed after *M. paratuberculosis* challenge, and a continuous trial, in which ATCC 334 was fed before and after *M. paratuberculosis* challenge. In the therapeutic treatment trial, groups of mice (*n* = 10) were orally inoculated with 10^9^ CFU/mouse of *M. paratuberculosis* on two consecutive days. At 6 weeks post infection mice began to receive daily doses of ATCC 334 resuspended in water given *ad libitum*. Water intake was monitored daily for each group of mice to ensure LAB inoculums of approximately 10^9^ CFU/mouse. Mice (*n* = 3–6) were sacrificed at 12 and 24 weeks post infection and the liver, spleen, mesenteric lymph node, and intestines were collected for bacterial, histopathological, and immunological examinations.

For the preventative and continuous treatment trials, groups of mice (*n* = 10) were fed daily doses of 10^9^ CFU of ATCC 334 resuspended in their water bottle for 1 month prior to oral infection with 10^9^ CFU/mouse *M. paratuberculosis* on two consecutive days. After *M. paratuberculosis* infection, half of the mice received water with no ATCC 334 (preventative treatment), while the other half continued receiving ATCC 334 resuspended in their water bottle for the reminder of the experiment (continuous treatment). All mice were sacrificed at 12 weeks post infection and tissues were collected for bacterial, histopathological, and immunological examinations.

### Cytokine analysis

At time of sacrifice, one third of the spleen was kept on ice before harvesting splenocytes for cytokine analysis (Wu et al., 2007). Briefly, spleens were sectioned and mashed through a mesh screen using the rubber plug of a syringe in a 60 mm Petri dish (CORNING, Corning, NY) in RPMI (Sigma-Aldrich, St. Louis, MO) supplemented with 1% fetal bovine serum (FBS) (Sigma-Aldrich), 1% L-glutamine (Gibco, Life Technologies), and 1% penicillin/streptomycin (Cellgro, Manassas, VA). Single cell suspensions were collected and pelleted by centrifugation followed by red blood cells lysis with 0.83% NH4Cl. Debris was allowed to settle before decanting mixture to a new tube. Cells were pelleted and resuspended in RPMI supplemented with 10% FBS, 1% L-glutamine, 1% pen/strep, and 1% non-essential amino acids (Gibco, Life Technologies) before seeding into 96 well flat bottom plates (Falcon, Franklin Lakes, NJ) at a density of 10^6^ cells/well. Splenocytes were supplemented with 100 U/ml of interlukin-2 (BD Pharmingen) and stimulated with either 10 μg/ml of Johnin purified protein derivative (PPD) (Animal and Plant Health Inspection Services, NVSL, Ames, IA), or 10 μg/ml of a positive control, Concanavalin A (Sigma-Aldrich). Stimulation doses used in this study were based on results from preliminary studies to optimize cytokine responses (data not shown). There were also non-stimulated, naive controls. All samples were cultured in duplicate for 48 h in a water-jacketed incubator (Thermo Scientific, Waltham, MA) at 37°C with 5% C0^2^. After 48 h, cell cultures were transferred to 96 well round bottom plates (Nunc, Roskilde, Denmark) and pelleted. Supernatants were collected at stored at −20°C until cytokine analysis.

Cytokine levels were measured using the Luminex TH1/TH2 Mouse 6-Plex Panel bead array kit as per the manufacturer instructions (Invitrogen, Frederick, MD). This kit quantified levels of interferon gamma (IFNγ), interlukin-2 (IL-2), interlukin-4 (IL-4), interlukin-5 (IL-5), interlukin-10 (IL-10), and interlukin-12 (IL-12) from splenocyte cell culture supernatants. Cytokine levels were determined by subtracting the background levels from each sample and averaged among the group (Suresh et al., 2005).

## DNA extraction and PCR

At time of sacrifice, tissue samples from the liver, spleen, mesenteric lymph node and intestines were collected and homogenized in PBS. Briefly, mycobacterial DNA was extracted from tissue samples through incubation with 5 mg/ml of lysozyme for 2 h at 37°C followed by an overnight incubation with 10% SDS and 2 mg/ml or proteinase-K at 56°C. The samples were then mixed with 0.4 volumes of 5 M potassium acetate, centrifuged, and supernatant was collected. DNA was precipitated using tris-saturated phenol:chloroform:isoamyl alcohol (25:24:1), followed by phenol removel with chloroform/isoamyl alcohol (24:1) and precipitation with3 M sodium acetate and 2.5 volumes of 100% ethanol. DNA was resuspended in ddH20. Polymerase chain reaction (PCR) was performed using the mycobacterial species-specific primers for the IS900 insertion sequences. Forward primer: TACCTTTCTTGAAGGGTGTTCGGGG, Reverse primer: TTGTGCCACAACCACCTCCG. PCR conditions: 94°C, 5 min, 30 × (94°C, 15 s, 60°C, 20 s, 72°C, 20 s) 72°C, 10 min, 4°C hold.

## Statistical analysis

One-Way ANOVA analysis was performed to evaluate differences in bacterial colonization in tissue samples and Luminex cytokine levels during the course of the study.

*P*-values of <0.05 were considered to be significant (SigmaPlot 11.0 software).

## Results

### *M. paratuberculosis* infection following oral delivery

In the first attempt to establish the oral model of murine infection, groups of mice were inoculated via oral gavage with 10^4^ CFU/animal or 10^5^ CFU/animal of *M. paratuberculosis* on two consecutive days. Clinically, all animals' survived oral infection and daily observations of infected animals showed no signs of illness throughout the duration of the study. All mice were sacrificed at 6 weeks post infection to quantify colonization levels in organs known to harbor *M. paratuberculosis* (liver, spleen, intestines and mesenteric lymph nodes). The 6 week time point was chosen based on clear colonization in the intraperitoneal model at this point in earlier studies (Shin et al., 2006; Scandurra et al., 2010). However, culturing of these organs showed no detectable levels of *M. paratuberculosis* (detection limit, 10 CFU/g) and all tissue PCR analyses were negative (data not shown).

**Figure 1:**
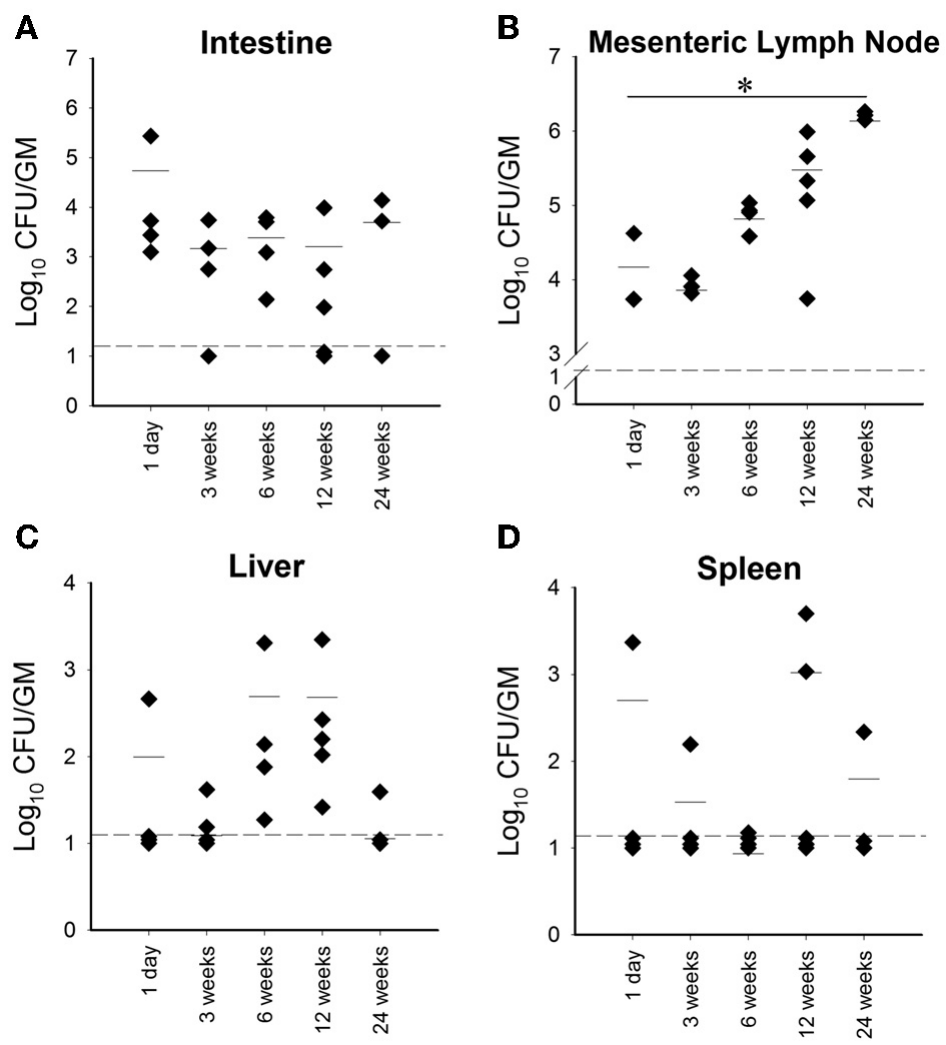
**Organ colonization from high dose orally infected mice**. Graphs **(A–D)** (**A**, Intestine; **B**, Mesenteric lymph node; **C**, Liver; **D**, Spleen) depict CFU/g obtained from plating serial dilutions of homogenized organs (intestines, mesenteric lymph node, liver and spleen) from mice orally infected with two consecutive doses of 10^9^ CFU of *M. avium* subsp. paratuberculosis K10 over a 24 week period. Each dot represents one mouse, the hash mark denotes the mean, and the dotted line represents the minimum detection limit. (^*^p < 0.05).

**Figure 2:**
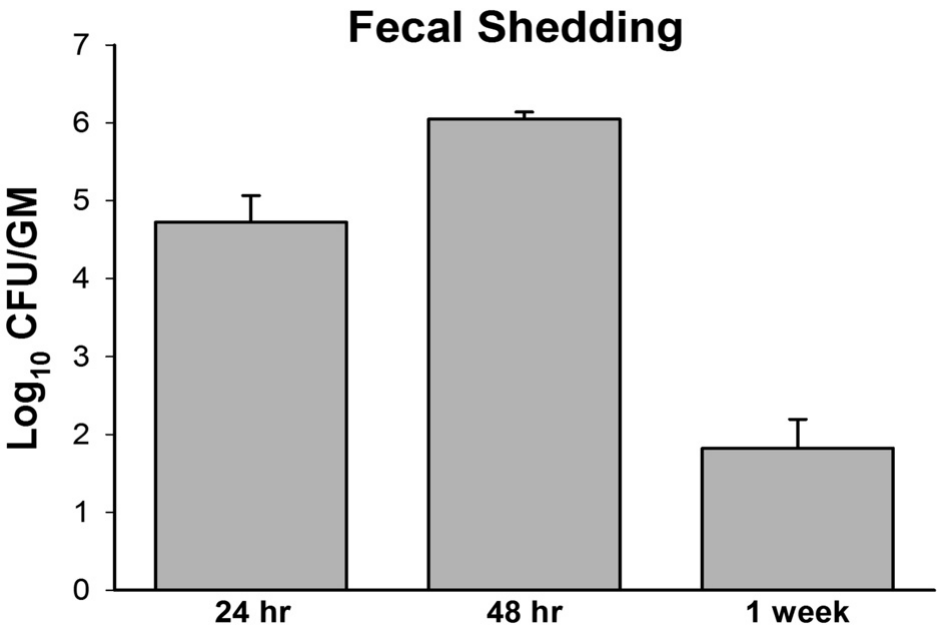
***M. avium* subsp. *paratuberculosis* shedding in mouse fecal samples**. Pooled fecal samples were collected from mice orally infected with two consecutive doses of 10^9^ CFU of M. avium subsp. paratuberculosis K10 Samples were decontaminated by HPC treatment and serial dilution plating on 7H10 agar was performed for quantification. All samples were run in duplicate.

In the second attempt, a higher dose, 10^9^ CFU/animal of *M. paratuberculosis* was used via oral gavage on two consecutive days. Challenged animals harbored detectable levels of bacteria as early as 1 day post infection from at least one organ per animal in each group; with the mesenteric lymph node and intestines showing colonization in all animals (Figure 1). After 3 weeks post infection, there was an overall decrease in colonization in cultured organs compared to day 1. Importantly, *M. paratuberculosis* was detected in the mesenteric lymph node of all animals by 3 weeks post infection. In addition, *M. paratuberculosis* was detected in the liver and spleen, suggesting that the bacteria had disseminated to several body organs following oral inoculation. By 6 weeks post infection, *M. paratuberculosis* showed an increase in bacterial load in the mesenteric lymph node as well as in the liver and intestines. Interestingly, none of the animals at this time point had detectable levels of infection in the spleen (Figure 1D). At 12 weeks post infection, *M. paratuberculosis* colonization remained at levels similar to earlier time point (6 weeks post infection) for the intestines and the liver, whereas the mesenteric lymph node had increased levels of colonization. The spleens at 12 weeks post infection showed detectable levels of *M. paratuberculosis* for two of the five animals observed. By the final time point of 24 weeks post infection, the liver and spleen showed an overall further decline in colonization, possibly due to the start of bacterial clearance, whereas the intestinal colonization remained steady and the mesenteric lymph node increased significantly (*p* < 0.05), reflecting its importance in establishing persistent infection. Fecal shedding of *M. paratuberculosis* was also monitored over the duration of the study. Results showed that within 24 h after receiving their first dose, mice were shedding approximately 10^4^ CFU per gram of feces (Figure 2). By 48 h, the mice received their second oral dose of *M. paratuberculosis* which further increased the bacterial load in the feces. However, the bacterial content greatly decreased by 1 weeks post infection, and was not detectable at any other point for the remainder of the study by culture or PCR (data not shown).

### Immune responses to *M. paratuberculosis* oral infection

**Figure 3:**
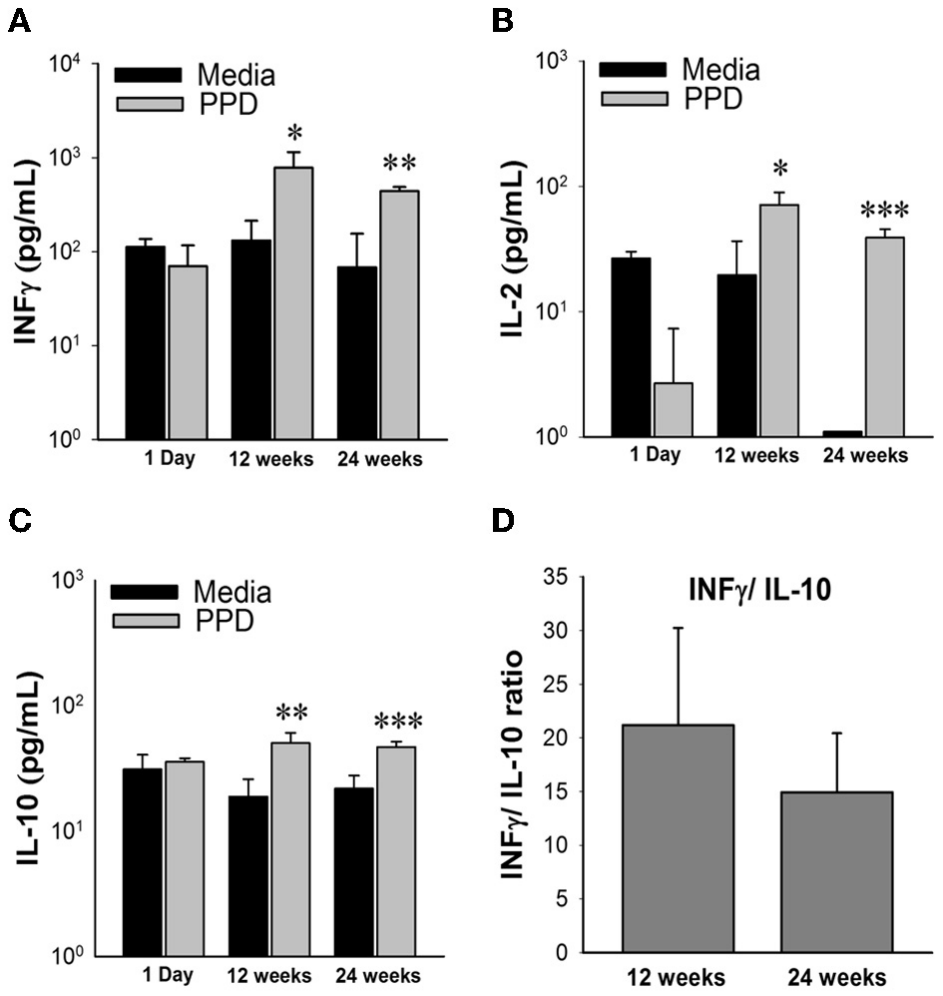
**Cytokine profiles of high dose *M. avium* subsp. *paratuberculosis* orally infected mice over 24 weeks post infection**. Graphs (**A–C**) depict cytokine levels of mice orally infected with two consecutive doses of 10^9^ CFU of *M. avium* subsp. paratuberculosis K10 over a 24 week period. At time of sacrifice, mouse spleens were collected and splenocytes were isolated and stimulated with Johnin Purified Protein Derivative (PPD) or media only for 48 h. Supernatant was collected and used for cytokine quantification by luminex bead array. Black bars represent the mean levels of media only background levels with their standard deviation and gray bars represent the mean levels from stimulation with PPD with their standard deviation. Significant increases in PPD stimulated splenocytes were determined by comparison to media only levels. (^*^p < 0.05, ^**^p < 0.01, ^***^p < 0.001). To determine shifts in immune response from 12 to 24 weeks, levels of IFNγ were divided by IL-10 from individual mice **(D)**. The mean ratio and standard deviation for each time point is shown.

To determine the host response to *M. paratuberculosis* infection, a representative group of TH_1_ (IFN-γ, IL-2, IL-12) and TH_2_ (IL-4, IL-5, IL-10) cytokines were analyzed from splenocytes collected from each animal at time of sacrifice and incubated in media supplemented with Johnin PPD for 48 h. ELISA-based cytokine profiling revealed that animals challenged with low dose (10^4^ CFU/animal, and 10^5^ CFU/animal) concentrations of *M. paratuberculosis* had no antigen-directed cytokine responses (data not shown). On the other hand, the high dose group (10^9^ CFU/mouse), showed significant release of IFN-γ (*p* = 0.03), IL-2 (*p* = 0.02), and IL-10 (*p* = 0.01) in harvested spleenocytes stimulated with Johnin compared to media only controls by 12 weeks post infection. By 24 weeks post infection, the TH_1_ cytokines, IFN-γ and IL-2, showed a decrease in overall levels compared with 12 weeks post infection, but they were still significantly upregulated from controls (*p* = 0.002, *p* = 0.0004). Unfortunately, release of IL-4, IL-5, and IL-12 cytokines in response to PPD was too low to be quantified at all time points. Interestingly, IL-10 (representative of TH_2_ cytokines) remained relatively constant and still significantly up regulated from the control (*p* < 0.01) at both 12 and 24 weeks post infection. Finally, we attempted to examine the shift from TH_1_ to TH_2_ type of cytokines by estimating IFN-γ/ IL-10 ratios. Interestingly, the TH1 responses were dominant throughout the examined points despite the TH_1_ response declined by 24 weeks post infection compared to earlier time point at 12 weeks post infection (Figure 3). Thus, oral infection induced antigen-specific immune responses, inducing both TH_1_ and TH_2_ *M. paratuberculosis* specific cytokines.

## Pathology of *M. paratuberculosis* following oral infection

**Figure 4:**
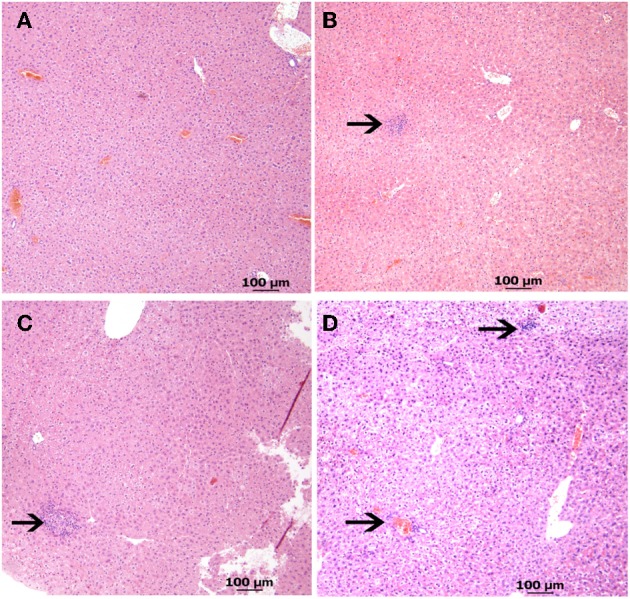
**Liver pathology from high dose oral *M. avium* subsp. *paratuberculosis* K10 challenge at various time points**. Images **(A–D)** reveal liver pathology from mice orally infected with two consecutive doses of 10^9^ CFU of *M. avium* subsp. paratuberculosis K10 over a 24 week period. H&E stained sections with 40 × magnification (scale bar = 100 μm) are shown. Images of 6 and 12 weeks post infection show lymphocyte inflammation and the image at 24 weeks post infection shows granulomatous inflammation. **(A)** Non-infected **(B)** 6 weeks **(C)** 12 weeks **(D)** 24 weeks.

Histological evaluation of the liver, spleen, and intestines from the low dose infection group at 6 weeks post infection revealed normal pathology and no acid-fast bacterium detected in any of the organs. The high dose *M. avium* subsp. *paratuberculosis* oral inoculation model, like other mouse models, showed histological lesions mostly affecting the liver, similar to those observed following intraperitoneal inoculation (Shin et al., 2006). Examining liver sections at 6 weeks post infection revealed normal histology in two of the four animals, while the remaining two showed early stages of lymphocyte inflammation (Figure 4). At 12 weeks post infection, liver sections revealed only one animal with undetectable lesions; while the remaining animals showed lesions ranged from early stages of lymphocyte aggregation to early stages of granuloma formation. By 24 weeks post infection, the granuloma formation in the liver sections was more prominent (Figure 4). Overall, the percentage of animals showing granulomatous inflammation went from 20% at 12 weeks post infection to 66% at 24 weeks post infection. All animals had normal pathology in the intestines and spleens at all time points. No acid fast bacteria were detected in any of the organs by Ziehl-Neelsen staining.

### The effect of lab ATCC 334 on *M. paratuberculosis* colonization

**Figure 5:**
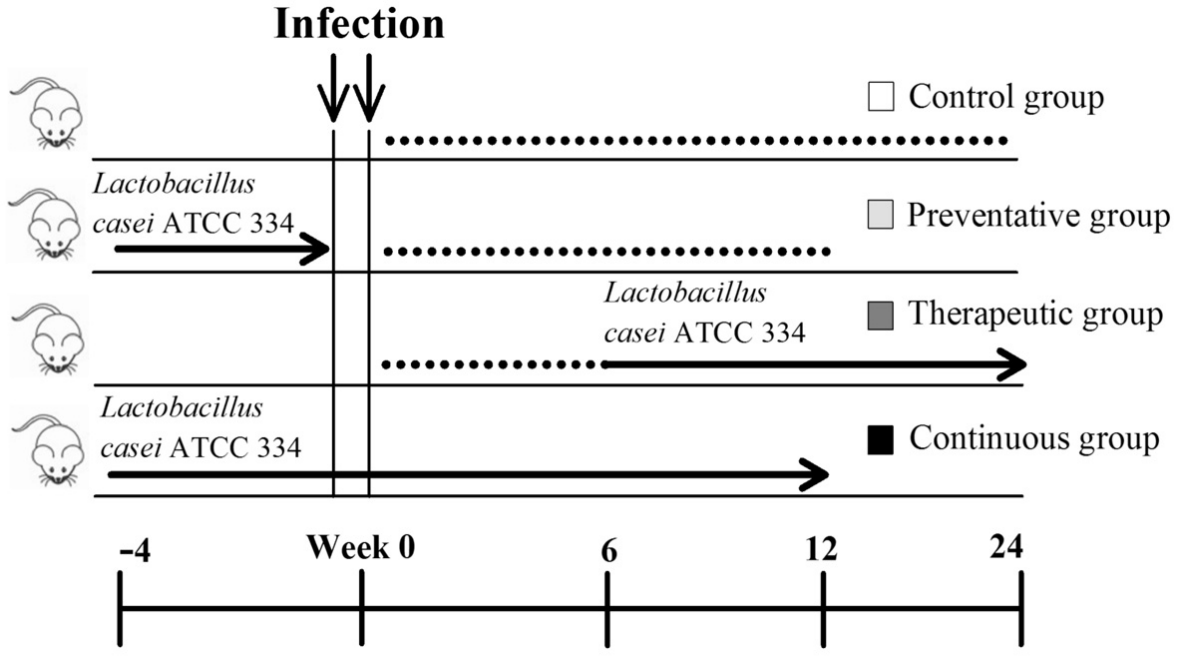
**Experimental design of *Lactobacillus casei* ATCC 334 feedings**. Three *Lactobacillus casei* ATCC 334 trials were conducted (preventative, therapeutic and continuous) along with a control. All mice were inoculated with two doses of 10^9^ CFU of *M. avium* subsp. *paratuberculosis* K10 on two consecutive days. The blue arrows represent the period of ATCC 334 feeding, where mice received daily doses of ~10^9^ CFU of ATCC 334. The black lines denote the parts of the trial period where no ATCC 334 was given. Mice were sacrificed and samples for colonization, histopathology and immunology were collected at 12 and/or 24 weeks post infection.

Because of our interest (and others) in developing an effective control strategy against Johne's disease, we evaluated the efficacy of the LAB strain, ATCC 334 as a tool to control *M. paratuberculosis* infection. We conducted a series of trials in order to determine the optimum feeding protocol to reduce colonization levels of *M. paratuberculosis* in mice organs. Our trials included preventative (feeding LAB before challenge with *M. paratuberculosis*), therapeutic (feeding LAB after challenge), and continuous (feeding LAB before and after challenge) groups, in comparison to a control group of mice challenged with *M. paratuberculosis* that did not receive any LAB ATCC 334 (Figure 5). Only the high dose (10^9^ CFU/mouse) of oral delivery was used in these trials. In addition, we tested the survival of LAB ATCC 334 at room temperature water before starting the feeding trials to ensure LAB viability during the feeding trials. As expected, the LAB ATCC 334 numbers remained constant for 2 days before dropping two logs on day 3 (data not shown). Accordingly, we replenished the culture resuspended in water every 2 days throughout the course of the study.

**Figure 6:**
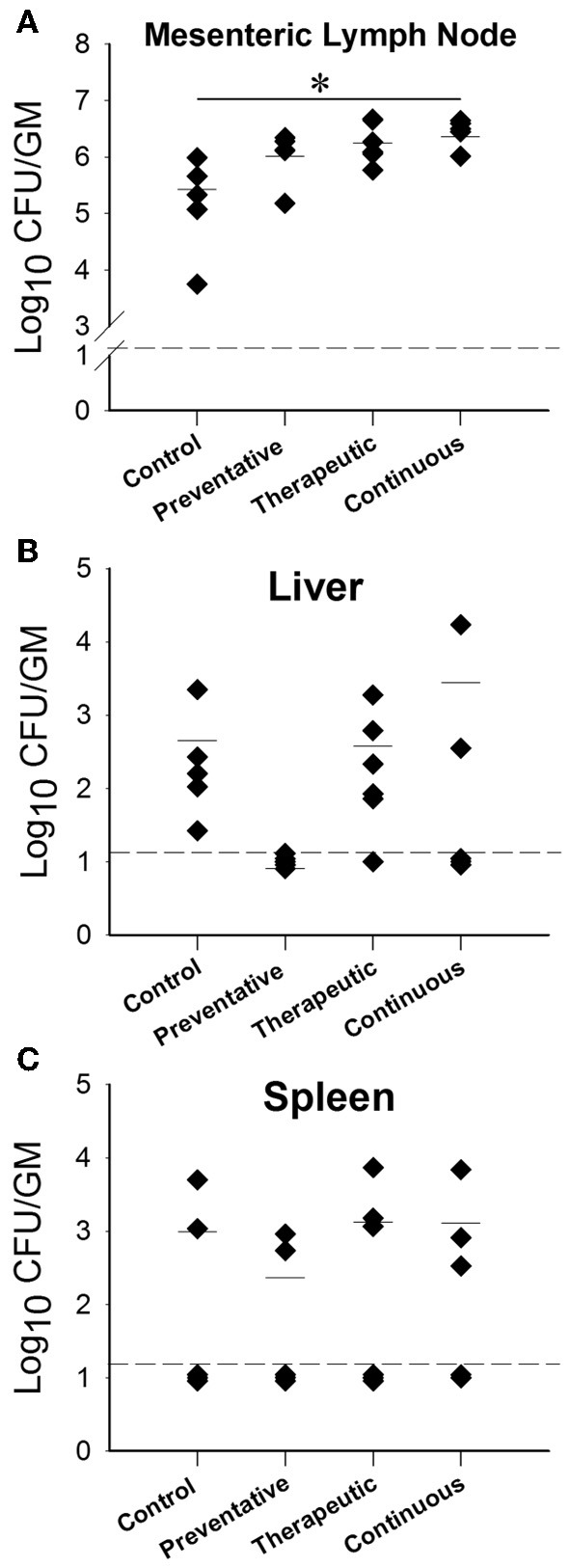
***M. avium* subsp. *paratuberculosis* organ colonization from different *Lactobacillus casei* ATCC 334 treatments at 12 weeks post oral *M. avium* subsp. *paratuberculosis* K10 infection**. Graphs **(A–C)** (**A**-Mesenteric lymph node, **B**-Liver, **C**-Spleen) depict the CFU/g of *M. avium* subsp. *paratuberculosis* obtained from the homogenized organs (mesenteric lymph node, liver and spleen) of mice treated with different ATCC 334 treatments and orally challenged with two doses of 10^9^ CFU *M. avium* subsp. *paratuberculosis* K10. Each dot represents one mouse, the hash mark denotes the mean, and the dotted line represents the minimum detection limit. (^*^p < 0.05).

An important readout of the oral model of paratuberculosis is the colonization level of *M. paratuberculosis* in mouse parenchymatous organs following different treatments with LAB. At 12 weeks post challenge (WPC), the mesenteric lymph node had increased *M. paratuberculosis* colonization levels in all LAB treatment groups compared to the control, however only the continuous treatment showed a statistically significant increase (Figure 6A). Interestingly, the liver of animals fed ATCC 334 prior to *M. paratuberculosis* challenge had no detectable levels of *M. paratuberculosis* (PCR analysis was negative, data not shown), unlike the control animals (Figure 6B). In addition, the *M. paratuberculosis* colonization levels were similar in the spleen of all treatment groups including the control (Figure 6C). Intestinal colonization at this time point was unable to be quantified due to overgrowth of LAB in all cultured plates. By 24 WPC, the therapeutic treatment group showed similar *M. paratuberculosis* colonization in all examined organs (Figure S1).

### ATCC 334 immunomodulation during *M. paratuberculosis* infection

**Figure 7:**
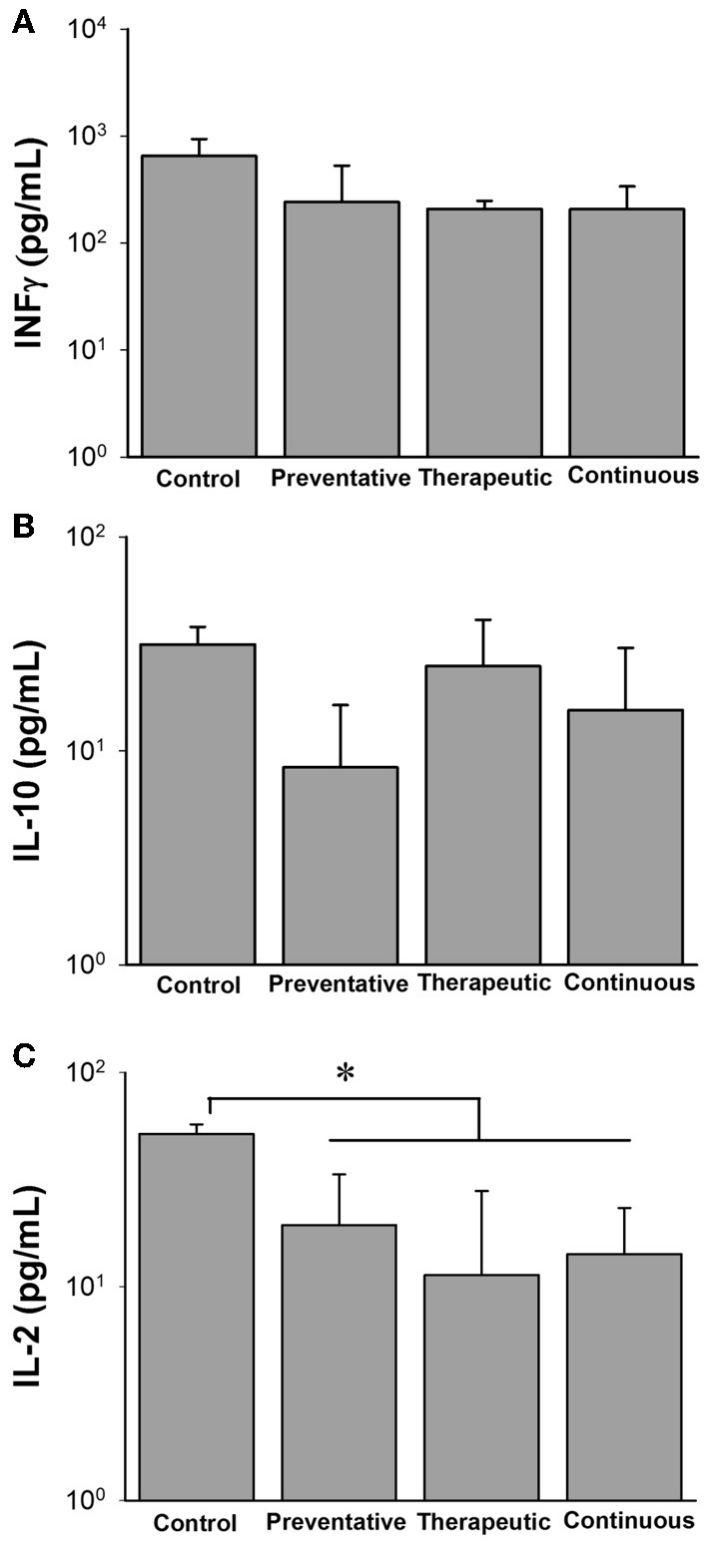
**Cytokine profiles of different Lactobacillus casei ATCC 334 treatments at 12 weeks post oral M. avium subsp. paratuberculosis K10 infection**. Graphs **A–C** (**A**. INFγ, **B**. IL-10, **C**. IL-12) depict cytokine levels of mice orally infected with two consecutive doses of 10^9^ CFU of *M. avium* subsp. paratuberculosis K10 and treated with different ATCC 334 treatments. At 12 weeks post infection, mouse spleens were collected and splenocytes were isolated and stimulated with Johnin Purified Protein Derivative (PPD) or media only for 48 h. Supernatant was collected and used for cytokine quantification by luminex bead array. Graph bars reflect the mean of each treatment group and their standard deviation. Levels were calculated by subtracting individual PPD stimulated levels from their background levels. Significant differences among groups are reflected by ^*^p < 0.05.

In addition to colonization data, we also evaluated the cytokine profiles for each of the different treatment groups in response to a mixture of *M. paratuberculosis* specific antigens (Johnin PPD). At 12 WPC, the inflammatory TH_1_ responses of IFN-γ were similar among all treatment groups, however only IL-2 levels were significantly reduced (Figure 7). Interestingly, the anti-inflammatory TH_2_ responses of IL-10 were reduced in the preventative treatment group but not to the statistical level. By 24 WPC, no differences in cytokine responses were detected between any of the treatment groups and the control (data not shown).

Finally, we utilized cytokine profiles to estimate TH_1_/TH_2_ ratios in order to determine if the LAB treatments had any effects on skewing the immune response toward cellular or humoral responses, respectively. At 12 WPC, the preventative ATCC 334 treatment skews the immune response more toward an inflammatory, TH_1_ response, while the therapeutic feeding regimen reduces the TH_1_ response (Figure S3). Interestingly, in the continuous group, the cytokine ratios fall between the individual levels for the preventative and therapeutic groups and were similar to the control. By 24 WPC, the TH_1_/TH_2_ ratio of the control and therapeutic groups revealed a complete shift from the responses seen at 12 WPC (Figure S2). The cytokine profile of the control group went from being skewed from a TH_1_ to a TH_2_ and the therapeutic group shifted from a predominant TH_2_ toward TH_1_, suggesting natural progression of paratuberculosis infection in the control group and immunomodulation favoring a protecting TH_1_ response from ATCC 334.

### The effects of lab treatments on the development of *paratuberculosis* lesions

To complete the evaluation of LAB treatments in the murine model of paratuberculosis, we evaluated the histology of tissues across the different treatment groups. By 12 WPC with *M. paratuberculosis*, the continuous group had the highest percentage (80%) of animals with normal pathology in the liver, followed by the preventative treatment group (60%) which also showed no granulomatous formation (Table 1). Interestingly, the therapeutic treatment group had liver pathology scores similar, or worse than that of the control group; 50% of the animals showed granuloma formation. By 24 weeks post challenge, the therapeutic group performed better than the control group, showing more animals with normal pathology and less with granulomatous inflammation, revealing a possible delayed protective response. Histology of the intestines and spleens from 12 and 24 weeks revealed normal pathology from all animals in the study. Taken together, these data suggests the importance of administering ATCC 334 prior to *M. paratuberculosis* infection.

**Table 1:** **Liver pathology results from different *Lactobacillus casei* ATCC 334 treatments at 12 and 24 weeks post oral *M. avium* subsp. *paratuberculosis* K10 infection**.

**Treatment 12 weeks**	**Normal**	**Lymphocytic inflammation**	**Granulomatous inflammation**
Control	1/5	3/5	1/5
Preventative	3/5	2/5	0/5
Therapeutic	1/6	2/6	3/6
Continuous	4/5	0/5	1/5
**Treatment 24 weeks**	**Normal**	**Lymphocytic inflammation**	**Granulomatous inflammation**
Control	1/3	0/3	2/3
Therapeutic	2/3	0/3	1/3

*Mice were orally inoculated with two doses of 10^9^ CFU of M. avium subsp. paratuberculosis K10 on two consecutive days from different ATCC 334 treatment groups (preventative, therapeutic, continuous and control). At 12 and/or 24 weeks post infection, mice were sacrificed and tissues pathology was examined. Each column represents the number of mice in that liver pathology category (normal, inflammation with a predominance of lymphocytes, granulomatous inflammation) over the total number of mice in that treatment group*.

## Discussion

Paratuberculosis is a chronic infectious disease that challenges the dairy industry and has no effective control strategy. Infection of dairy producing animals and the contamination of dairy products with *M. paratuberculosis* could increase the potential exposure of humans to *M. paratuberculosis* and the development of Crohn's disease (Chamberlin et al., 2001). Therefore, it is critical to develop a cost effective animal model that mimics the natural infection route and to investigate alternative treatment options. In this study, we optimized the oral model of murine infection with *M. paratuberculosis* to develop a better model for paratuberculosis. We also employed this model to evaluate immune-modulation of host responses using LAB ATCC 334, in reducing the impact of *M. paratuberculosis* infection using an assortment of feeding trials. Earlier reports indicated that low infectivity rates are the main drawback for the oral mouse model (Mutwiri et al., 1992; Veazey et al., 1995). As suggested before (Harris and Lammerding, 2001) in other oral challenge models for mice, higher doses of at least 10^8^ CFU/animal of *M. paratuberculosis* are needed to establish reproducible infection. In our hands, mice infected with low doses of 10^4^ CFU/animal and 10^5^ CFU/animal did not show any detectable levels of *M. paratuberculosis* by culture or PCR, and showed no antigen specific immune responses or pathological lesions. On the other hand, the challenge with high doses (10^9^ CFU/animal) yielded persistent infection, as indicated by culture, in at least one organ of all infected animals. By dosing orally, we were able to isolate *M. paratuberculosis* from fecal samples during the first few days post infection, most likely because of the direct passage through the GI tract. It is noteworthy to mention here that the observed persistence and replication of *M. paratuberculosis* in the mesenteric lymph node strongly suggest the importance of the lymph nodes in establishing chronic infection and represents a good target to track progression of paratuberculosis in the murine model. Interestingly, compared to other models of murine *M. paratuberculosis*, the oral route is more comparable to the natural infection, where bacterial burden is higher in the lymph node and intestines relative to the liver and spleen (Mutwiri et al., 1992; Siguroardottir et al., 2004) as shown before in other ruminants (e.g., goats and cows) (Corpa et al., 2000; LaMarca et al., 2004).

In addition to colonization data, we examined the host (immune and pathological) responses to *M. paratuberculosis* oral infection. Overall host responses were similar to the responses observed during *M. paratuberculosis* infection in ruminants, [increased levels of pro-inflammatory cytokines IFN-γ and IL-2 which are the essential cytokines for controlling the intracellular *M. paratuberculosis* pathogen early in infection (Coussens et al., 2004) and progression of infection toward the late subclinical stage where the pro-inflammatory TH_1_ response is diminished and an increase in anti-inflammatory IL-10 is seen (Coussens, 2001)]. This suggests that *M. paratuberculosis* oral infection in mice could be highly similar the progression of Johne's disease in ruminants.

Due to the success of the high dose inoculum, we chose this model to serve as the route of infection for our LAB feeding trials. We expected that mice receiving LAB ATCC 334 treatment would have reduced colonization, since previous studies have shown that LAB inhibits growth of *M. paratuberculosis* (Mariam, 2009; Van Brandt et al., 2011). Interestingly, continuous feeding of LAB ATCC 334 produced the opposite results, where the controls were colonized with *M. paratuberculosis* at levels significantly lower than the LAB fed groups, especially in the mesenteric lymph node. A possible hypothesis is that ATCC 334 stimulated the gut lymphocytes, as has been observed with *L. acidophilus* NCFM, thereby enhancing migration to the mesenteric lymph node, and providing new target cells for *M. paratuberculosis* infection and proliferation (personal communications, Steele). Because LAB strains have been shown to alter cytokine profiles in the presence of mycobacterium (Ghadimi et al., 2010), we examined the effect of LAB ATCC 334 in the murine oral model of paratuberculosis. Interestingly, the preventative trial TH_1_/TH_2_ ratio at 12 weeks showed how feeding ATCC 334 before *M. paratuberculosis* infection skewed the immune response toward a protective TH_1_ response and resulted in less histopathology than the control or therapeutic feedings. This improvement in histopathology is most likely due to strain specific immunomodulation of anti-inflammatory IL-10 secretion (Hall and Ratledge, 1984; Corr et al., 2009). Lower levels of IL-10 production are correlated with better control of *M. paratuberculosis* infections (Murray, 1999). However, at 12 WPC in the therapeutic trial, the opposite trend was observed. Conversely, by 24 WPC, a shift in the TH_1_/TH_2_ ratio toward the inflammatory response was seen in the animals receiving therapeutic ATCC 334 compared to the control group. This shift reflects a delayed response in the LAB-treated groups with the potential for a long-term immunomodulatory property. Finally, an investigation of the changes in intestinal microbiota of LAB treated animals would increase our understanding of the mechanism of action of the used LAB strain.

In conclusion, there are several benefits of having a well-established animal model for *M. paratuberculosis* challenge and vaccine studies. The developed oral model described in this study indicated that two consecutive *M. paratuberculosis* oral gavage dosing of greater than 10^9^ CFU per animal resulted in a reproducible model of paratuberculosis in mice with similarity to the Johne's disease development in ruminants. Additionally, feeding the LAB ATCC 334 could have favorable immunomodulation and better pathology if they administrated before infection with *M. paratuberculosis*. Although differences were observed between controls and ATCC 334 treatment groups, further studies investigating long-term immunomodulatory properties of LAB are needed as well as screening additional LAB strains that can positively affect colonization.

Although mice and cattle have profound differences in GI anatomy and microbial populations, this study acts as a preliminary screening measure that can be used with mice before testing in a ruminant model, since other parameters of infection, such as cytokine responses and colonization accurately reflect paratuberculosis disease progression. Overall, there is great potential for the use of LAB as a control option against paratuberculosis; not only as feed additives, but also as vaccine carriers (Mierau et al., 2005). However, care must be taken for selection of the appropriate strain and regimen of LAB treatments in dairy operations affected by Johne's disease.

### Conflict of interest statement

The authors declare that the research was conducted in the absence of any commercial or financial relationships that could be construed as a potential conflict of interest.

## Supplementary material

Figure S1 ***M. avium* subsp. *paratuberculosis* organ colonization from therapeutic *Lactobacillus casei* ATCC 334 treatments at 24 weeks post oral *M. avium* subsp. *paratuberculosis* K10 infection**.

Figure S2 **Cytokine profiles of therapeutic *Lactobacillus casei* ATCC 334 treatment versus control at 24 weeks post oral *M. avium* subsp. *paratuberculosis* K10 infection**.

Figure S3 **IFNγ/IL-10 ratios for different *Lactobacillus casei* ATCC 334 treatments vs. control at 12 and 24 weeks post *M. avium* subsp. *paratuberculosis* infection**.
