# Supplementary material for: A murine oral model for Mycobacterium avium subsp. paratuberculosis infection and immunomodulation with Lactobacillus casei ATCC 334
Source: Front Cell Infect Microbiol. 2014 Feb 5;4:11. doi: 10.3389/fcimb.2014.00011 (PMC3914629; doi:10.3389/fcimb.2014.00011)
Supplement: Figure S1 — M. avium subsp. paratuberculosis organ colonization from therapeutic Lactobacillus casei ATCC 334 treatments at 24 weeks post oral M. avium subsp. paratuberculosis K10 infection. [file Presentation1.PDF]

SUPPLEMENTARY MATERIAL:

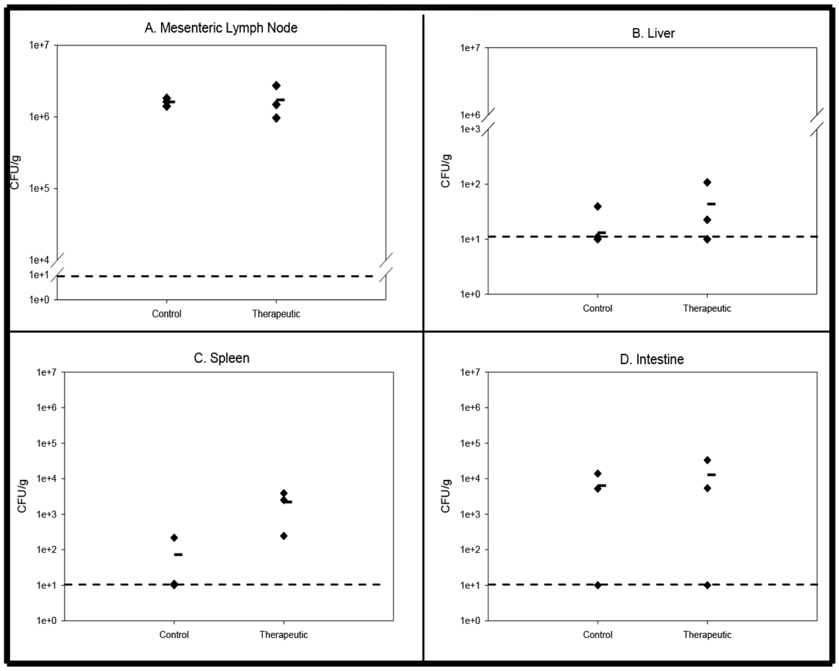

Figure S1. *M. avium* subsp. *paratuberculosis* organ colonization from therapeutic *Lactobacillus casei* ATCC 334 treatments at 24 weeks post oral *M. avium* subsp. *paratuberculosis* K10 infection. Graphs A-D (A-Mesenteric lymph node, B- Liver, C-Spleen, D-Intestine) depict the CFU/g of *M. avium* subsp. *paratuberculosis* obtained from homogenized organs (mesenteric lymph node, liver, spleen, and intestine) of mice treated with ATCC 334 after oral challenge with 10^9 CFU *M. avium* subsp. *paratuberculosis* K10. Each dot represents one mouse, the hash mark denotes the mean, and the dotted line represents the minimum detection limit.

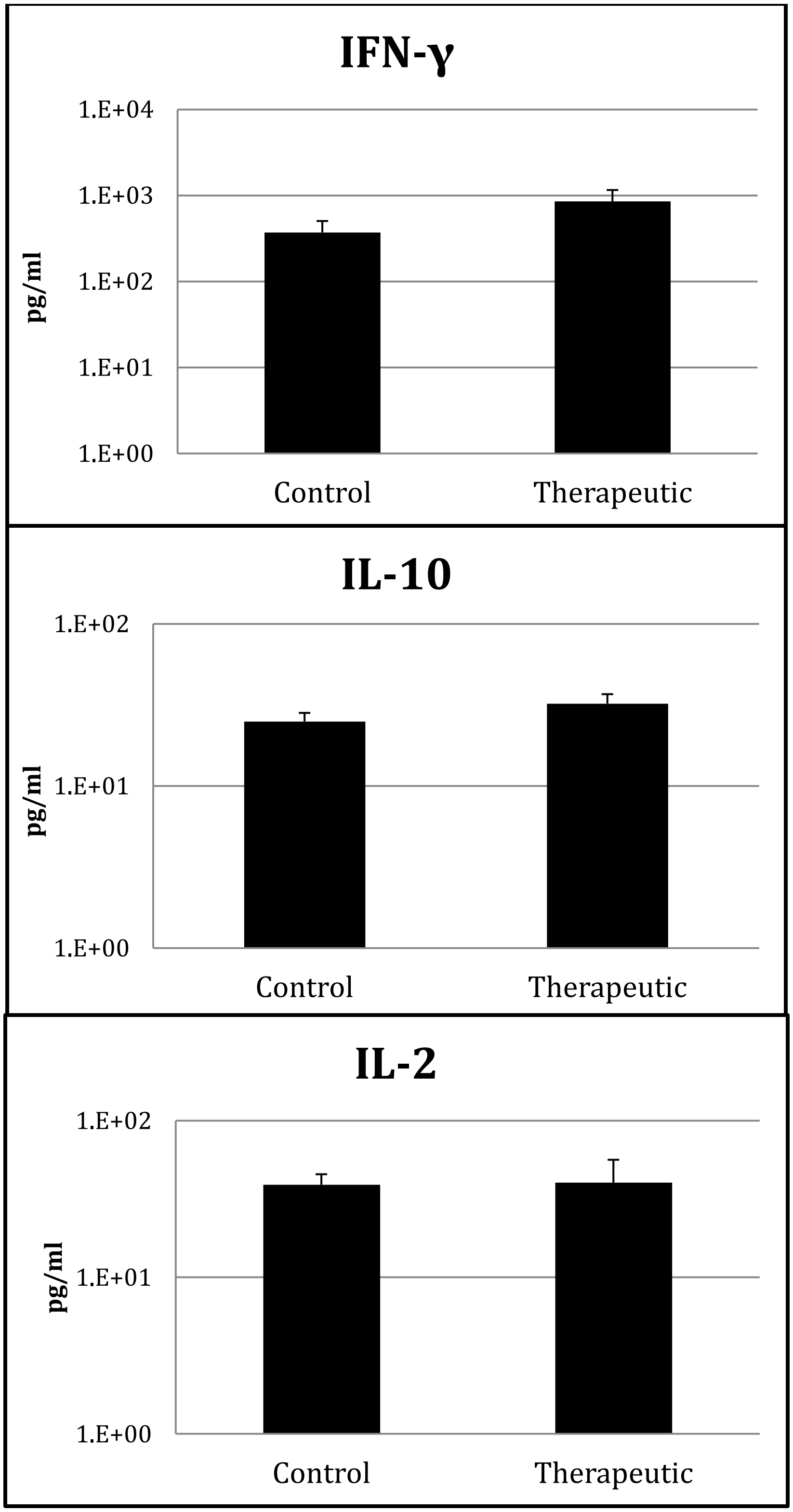

Figure S2. Cytokine profiles of therapeutic *Lactobacillus casei* ATCC 334 treatment versus control at 24 weeks post oral *M. avium* subsp. *paratuberculosis* K10 infection. Graphs depict

cytokine levels of mice orally infected with two consecutive doses of 10^9 CFU of *M. avium* subsp. *paratuberculosis* K10 and treated with ATCC 334 for 24 weeks post infection. At time of sacrifice, mouse spleens were collected and splenocytes were isolated and stimulated with Johnin Purified Protein Derivative (PPD) or media only for 48 hours. Supernatant was collected and used for cytokine quantification by luminex bead array. Graph bars reflect the mean of each treatment group and their standard deviation. Levels were calculated by subtracting individual PPD stimulated levels from their background levels. Significant differences among groups are reflected by *= $p < 0.05$.

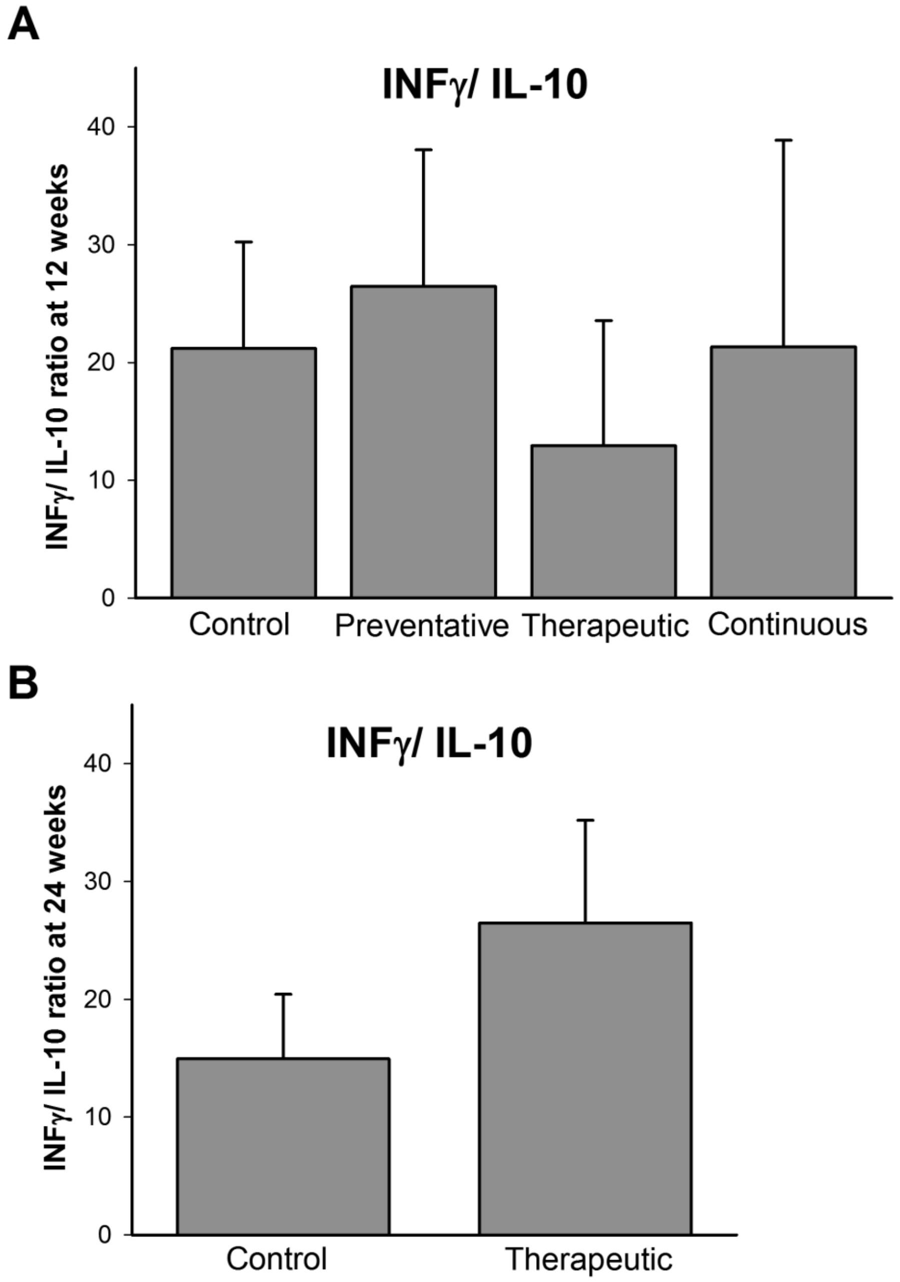

Figure S3. IFNγ/ IL-10 ratios for different *Lactobacillus casei* ATCC 334 treatments versus control at 12 and 24 weeks post *M. avium* subsp. *paratuberculosis* infection. Graphs depict mean IFNγ/ IL-10 ratios and their standard deviation for mice orally infected with two consecutive doses of 10^9 CFU of *M. avium* subsp. *paratuberculosis* K10 and treated with ATCC 334 for 12

and/or 24 weeks post infection. Values were calculated by dividing the levels of IFNγ by IL-10 from individual mice in each treatment group**.** The mean ratio and standard deviation for each time point is shown.
